# Dr. Orrin T. Stacey

**Published:** 1917-04

**Authors:** 


**Dr. Orrin T. Stacey,** Buffalo 1860, died Feb. 21 at his home in Rochester, aged 82. He was born in Centerville, Allegany Co., N. Y., in 1835. He taught school for several years, read medicine in the office of his father, Wm. A. Stacy, who was a graduate of the Castleton, Vt., Medical College, and settled in Rushford, where he practiced medicine for 25 years, moving to Rochester in 1885, practicing there till 1893 when he established a candy business which has grown to large proportions. He was a republican member of the state legislature in 1875 and 1876. He also developed considerable tracts of real estate in Rochester. In the spring of 1905 he underwent a severe operation at Johns Hopkins Hospital and never fully regained his health.

